# Inherited Variation in Cytokine, Acute Phase Response, and Calcium Metabolism Genes Affects Susceptibility to Infective Endocarditis

**DOI:** 10.1155/2017/7962546

**Published:** 2017-06-04

**Authors:** Anastasia V. Ponasenko, Anton G. Kutikhin, Maria V. Khutornaya, Natalia V. Rutkovskaya, Natalia V. Kondyukova, Yuri N. Odarenko, Yana V. Kazachek, Anna V. Tsepokina, Leonid S. Barbarash, Arseniy E. Yuzhalin

**Affiliations:** ^1^Research Institute for Complex Issues of Cardiovascular Diseases, Sosnovy Boulevard 6, Kemerovo 650002, Russia; ^2^Department of Oncology, CRUK/MRC Oxford Institute for Radiation Oncology, University of Oxford, Old Road Campus Research Building, Roosevelt Drive, Oxford OX3 7DQ, UK

## Abstract

Infective endocarditis (IE) is a septic inflammation of the endocardium. Recognition of microbial patterns, cytokine and acute phase responses, hemostasis features, and alterations in plasma lipid and calcium profile all have been reported to affect pathogenesis and clinical course of IE. Having recruited 123 patients with IE and 300 age-, sex-, and ethnicity-matched healthy blood donors, we profiled their genomic DNA for 35 functionally significant polymorphisms within the 22 selected genes involved in the abovementioned pathways, with the further genetic association analysis. We found that the G/A genotype of the rs1143634 polymorphism within the *IL1B* gene, the G/T genotype of the rs3212227 polymorphism within the *IL12B* gene, the A/G genotype of the rs1130864 polymorphism within the *CRP* gene, and the G allele of the rs1801197 polymorphism within the *CALCR* gene were associated with a decreased risk of IE whereas the T/T genotype of the rs1205 polymorphism within the *CRP* gene was associated with a higher risk of IE. Furthermore, heterozygous genotypes of the rs1143634 and rs3212227 polymorphisms were associated with the higher plasma levels of IL-1*β* and IL-12, respectively. Our results indicate that inherited variation in the cytokine, acute phase response, and calcium metabolism pathways may be linked to IE.

## 1. Introduction

Infective endocarditis (IE) is defined as an infection of the endocardial surface, which may involve native or prosthetic heart valves, the mural endocardium, interventricular septum, chordae tendineae, or surfaces of intracardiac devices [1]. In general, IE is caused by bacteria, in particular *Staphylococcus*, *Streptococcus*, and *Enterococcus* spp. [2], but fungi have also been reported as a culprit [3]. The most common signs and symptoms of IE are malaise, fatigue, coughing, chills, fever, weight loss, and heart murmur [1]. Incidence of IE significantly varies in different countries (from 1.5/100,000 in Netherlands to 11.6/100,000 in United States), with an average case fatality rate of 25% [4]. However, case fatality rates considerably depend on the etiological agent: from 10% in patients infected with *Streptococcus* spp. to 40% in those infected with *Staphylococcus aureus* [5–9]. The development and course of IE may depend on (1) the recognition of pathogen-associated molecular patterns as well as cytokine and acute phase response to causative agents [10–12], (2) hemostasis [13–15], (3) plasma lipid profile [16, 17], and (4) plasma calcium profile [18, 19].

Progress in genotyping technologies reasoned the studies on the association of single nucleotide polymorphisms (SNPs) with human diseases [20]. SNPs located in different genomic regions may cause distinct consequences, including altered (1) transcription initiation; (2) mRNA splicing; (3) protein folding, stability, and expression; and (4) posttranslational modifications [21]. Previously, we reported that the C/C genotype of the rs3775073 polymorphism within the *TLR6* gene is associated with a twofold decreased risk of IE; other polymorphisms within the genes encoding pattern recognition receptors did not show a predictive value. Here, we investigated whether the SNPs within the cytokine, acute phase response, lipid metabolism, and calcium metabolism genes can alter individual susceptibility to IE.

## 2. Materials and Methods

### 2.1. Population

Inclusion criteria were as follows: (1) living in Kemerovo region for ≥2 generations; (2) Russian ethnicity; (3) clinically (modified Duke criteria, at least 1 major and 1 minor criteria or 3 minor criteria being fulfilled [22]) and histologically verified diagnosis of IE; and (4) written informed consent. Exclusion criteria were as follows: (1) belonging to the immigrant or aboriginal populations, (2) previous cancer diagnosis, (3) concomitant mental disorders and/or autoimmune diseases, (4) drug addiction, and (5) refusal to sign a written informed consent. All the patients underwent antibiotic therapy in the acute phase during the first admission to their district hospital according to the European guidelines [22]. Antibiotic therapy and treatment of all concomitant diseases were further performed during the preoperative period at the Kemerovo Cardiology Centre.

In total, we recruited 161 patients with IE admitted to our Research Institute during 2009–2016. After exclusion of 38 patients due to the abovementioned criteria, the study group finally included 123 patients (Table 1). The control group for this study was formed from 300 age- (±6 years), sex-, and ethnicity-matched asymptomatic blood donors with no history of drug addiction, cardiovascular disease, cancer, and autoimmune and mental disorders (Table 1). Data on the clinicopathological features and hospital complications/case fatality rate of patients with IE are presented in Table 2. The local ethical committee of the Research Institute for Complex Issues of Cardiovascular Diseases approved the study protocol. All the participants provided written informed consent after the study was fully explained.

### 2.2. SNP Selection and Genotyping

For this study, we defined four main criteria for SNP selection: (1) location within cytokine, acute phase response, hemostasis, lipid metabolism, or calcium metabolism genes; (2) minor allele frequency ≥ 5% for Russian population tested with HapMap; (3) functional alteration of protein expression; and (4) few or no studies investigating the role of an SNP in IE. The National Center for Biotechnology Information dbSNP, SNPinfo, and SNPnexus databases were utilized for the SNP selection [23, 24]. In total, we selected 35 SNPs within 22 genes (Table 3).

The procedures of DNA extraction and genotyping were the same as previously described [12, 25–27]. Briefly, 5 mL of venous blood was collected into a tube with ethylenediaminetetraacetic acid. Then, 0.5 mL of blood was mixed with 1 mL of saline-sodium citrate buffer (Promega) following centrifugation at 12,000 rpm for 2 min. The pellet was digested in a mixture of 10% sodium dodecyl sulfate (Sigma) with 100 *μ*g/mL proteinase K (Helicon) for 3 h at 50°C. Upon digestion, we added phenol : chloroform : isoamyl alcohol (25 : 24 : 1) to the lysate, vortexed it for 20 seconds, and centrifuged at 12,000 rpm for 15 min; 70% ethanol was further utilized to precipitate genomic DNA from a viscous interphase layer. The sample was finally centrifuged at 12,000 rpm for 5 min. DNA pellet was incubated overnight in deionized water at room temperature and was further stored at −70°C until use.

**Figure 1:**
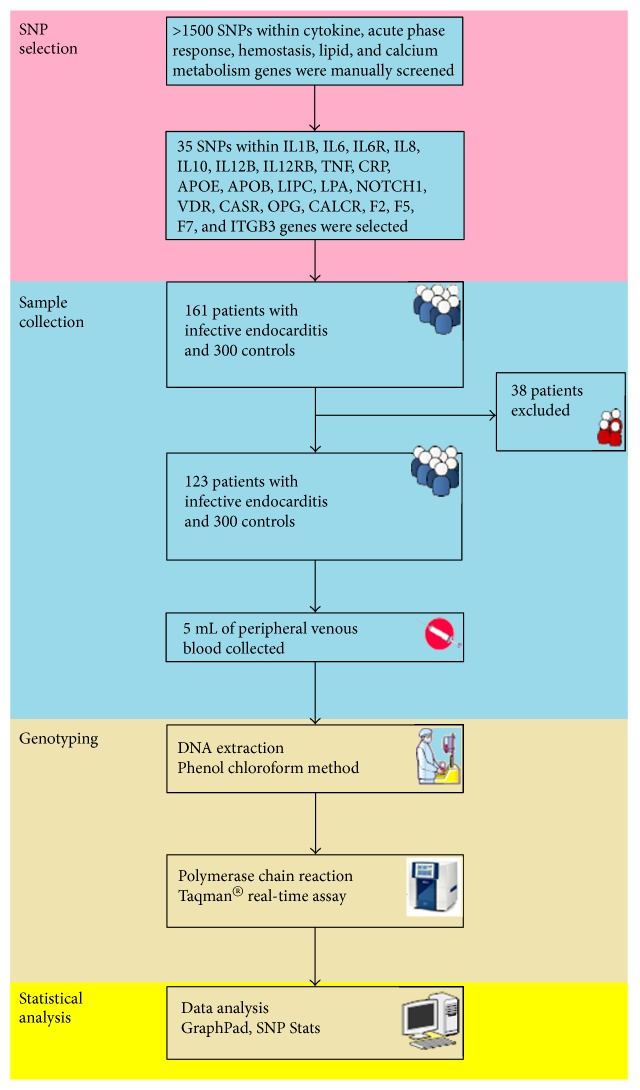
Study workflow.

Genotyping was carried out in a 96-well format using the TaqMan SNP assay on the ViiA™ 7 Real-Time PCR System (Life Technologies) according to the manufacturer's instructions. Amplification mixture contained 100 ng of DNA, 1.25 *μ*L of each primer, 2.5 mM of MgCl2, 1 mM of dNTPs, and 1 U of Taq polymerase (Life Technologies), in a total volume of 10 *μ*L. We employed the following polymerase chain reaction (PCR) protocol: hold stage 50°C for 120 s and 95°C for 10 min and PCR stage 95°C for 15 s and 60°C for 1 min repeated in 40 cycles. Table 3 demonstrates the sequence-specific primers for the genotyped SNPs. Laboratory staff was blinded to patient status, and one-tenth of the samples was repeatedly genotyped for quality control purposes. The study workflow is summarized in Figure 1.

### 2.3. Measurement of Plasma Cytokine Level

Venous blood was withdrawn during hospital admission and 7 days postoperation. The plasma was obtained with a centrifugation for 15 min at 1780 ×g and −4°C; 300 *μ*L aliquots have been stored at −80°C until use. The plasma levels of interleukin- (IL-) 1*β*, IL-6, IL-8, IL-10, IL-12, tumor necrosis factor- (TNF-) *α*, and C-reactive protein (CRP) were measured by enzyme-linked immunosorbent assay using the kits purchased from eBioscience (BMS224/2, BMS213/2, BMS204/3CE, BMS215/2, BMS238CE, BMS223/4CE, and 88-7502-28, resp.) according to the manufacturer's instructions. All samples were plated in duplicates, with average concentrations used for further analysis.

### 2.4. Statistical Analysis

The statistical analysis was performed as in [12, 25–27] using GraphPad Prism (GraphPad Software) and SNPStats, a web tool for the analysis of genetic association studies [28].

## 3. Results

Here, we evaluated the distribution of SNPs within 22 core genes involved in cytokine activity, acute phase response, lipid metabolism, and calcium metabolism genes in a sample of 123 patients with IE and 300 matched asymptomatic control individuals (Table 3). Tables 1 and 2 summarize demographic and clinicopathological characteristics of cases and controls. We did not find statistically significant gender differences between the groups (Table 1). The genotype distributions in both groups are presented in Table 4. All the genotype distributions were in Hardy-Weinberg equilibrium that confirmed a good quality of the genotyping.

We first asked whether an inherited variation within the genes encoding cytokines and acute phase proteins may play a role in the risk of IE development. Having performed the genetic association analysis with the adjustments for age and gender, we found that the G/A genotype of the rs1143634 polymorphism within the *IL1B* gene was associated with a lower risk of IE (OR = 0.43, 95% CI = 0.26–0.71, *p* = 0.0016, overdominant model, Table 4). Similarly, the G/T genotype of the rs3212227 polymorphism within the *IL12B* gene correlated with a decreased risk of IE (OR = 0.57, 95% CI = 0.34–0.94, *p* = 0.0250, overdominant model, Table 4). Finally, we observed that the A/G genotype of the rs1130864 polymorphism within the *CRP* gene was also associated with a lower risk of IE (OR = 0.54, 95% CI = 0.34–0.86, *p* = 0.0083, overdominant model, Table 4). Conversely, the T/T genotype of the rs1205 polymorphism within the *CRP* gene was associated with a higher risk of IE (OR = 2.42, 95% CI = 1.32–4.43, *p* = 0.0047 according to a recessive model, Table 4). Other SNPs were not significantly different between cases and controls (Table 4).

We further investigated whether the inherited variation in the pathways of hemostasis, lipid metabolism, and calcium metabolism can be linked to IE. The A/G genotype of the rs13290979 polymorphism within the *NOTCH1* gene and the G allele of the rs1801197 polymorphism within the *CALCR* gene were associated with a lower risk of IE (OR = 0.54, 95% CI = 0.34–0.84, *p* = 0.0062 according to an overdominant model; OR = 0.56, 95% CI = 0.38–0.82, *p* = 0.0020 according to a log-additive model, resp., Table 4). Other SNPs were not significantly associated with IE (Table 4).

**Figure 2:**
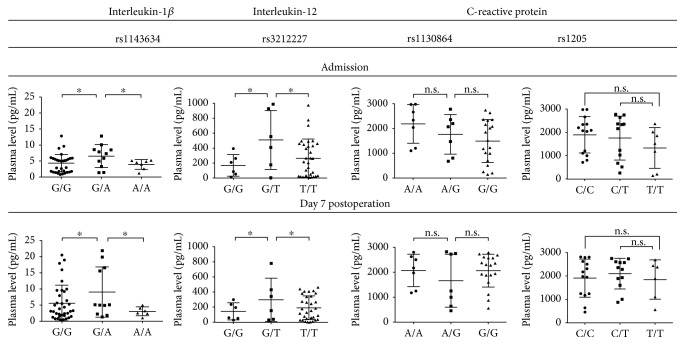
Measurement of plasma interleukin-1*β*, interleukin-12, and C-reactive protein levels in patients with infective endocarditis at the hospital admission and 7 days postoperation. Two-tailed Student's *t*-test with further Tukey's post hoc test to adjust for multiple comparisons; each dot is a measure from one patient, ^∗^*p* < 0.05, n.s. is for not significant.

As cytokines are easily detected in blood, we then sought to explore the functional consequences of four SNPs reaching the significance threshold in *IL1B*, *IL12*, and *CRP* genes. We evaluated the plasma levels of these proteins obtained from the patients with IE before admission and 7 days postoperation (Figure 2). The G/A genotype of the rs1143634 polymorphism within the *IL1B* gene and the G/T genotype of the rs3212227 polymorphism within the *IL12B* gene were associated with a higher plasma level of IL-1*β* and IL-12, respectively, at either time point (Figure 2). However, no significant associations were revealed for CRP (Figure 2). To further reinforce the associations between the marker SNPs, plasma cytokine levels, and risk of IE, we hypothesized that those SNPs which did not demonstrate a predictive value would not show associations with altered plasma levels of the corresponding proteins. Hence, we performed the similar analysis for the SNPs within the genes encoding cytokines which are generally elevated in patients with IE, that is, TNF-*α*, IL-6, IL-8, and IL-10 (Supplementary Figures 1 and 2 available online at https://doi.org/10.1155/2017/7962546). Expectedly, no statistically significant associations were found.

## 4. Discussion

Despite the recent progress in diagnosis and treatment [1, 2], the basis of genetic susceptibility to IE remains vaguely uncovered. Early investigation by Vollmer et al. revealed the G allele of the rs2232596 polymorphism and the T allele of the rs2232582 polymorphism within the *LBP* gene being associated with a higher risk of IE [29]. As known, lipopolysaccharide-binding protein is released into the bloodstream as an acute phase response protein during IE [19]. Further studies by Daga et al. [30, 31] and Durante-Mangoni et al. [32] did not find any association between the SNPs within the hemostasis genes (*PTH*, *FV*, *GPIb*, *GPIIIa*, and *FcγRIIa*) and IE. Our previous study identified the C/C genotype of the rs3775073 polymorphism within the *TLR6* gene as a protective factor [12] while a study by Bustamante et al. suggested the A allele of the rs5743708 polymorphism within the *TLR2* gene as a risk factor [33]. In this study, we selected 35 SNPs within 22 genes involved in the development of IE: *IL1B*, *IL6*, *IL6R*, *IL8*, *IL10*, *IL12B*, *IL12RB*, *TNF*, *CRP*, *APOB*, *APOE*, *LIPC*, *LPA*, *NOTCH1*, *VDR*, *CASR*, *OPG*, *CALCR*, *F2*, *F5*, *F7*, and *ITGB3*. Previous studies demonstrated the elevated serum levels of IL-1*β*, IL-6, IL-8, IL-10, IL-12, TNF-*α*, and CRP in patients with IE compared to the healthy blood donors which indicates the possible importance of these cytokines in the clinical course of IE; however, the exact role of these inflammatory molecules in IE remains elusive [11, 34]. Apolipoproteins B and E, lipoprotein (a), and hepatic lipase are involved in the metabolism of lipids that can be important for IE development since patients with IE have lower level of serum high-density lipoprotein cholesterol (HDL) than healthy blood donors; furthermore, low serum HDL level is indicative of a complicated IE course [16]. Another well-established risk factor of IE is heart valve calcification, with NOTCH1, vitamin D receptor, calcium-sensing receptor, osteoprotegerin, and calcitonin receptor being the major regulators of serum calcium and phosphorus levels [27, 35, 36]. Finally, patients with IE are prone to thromboembolism due to increased systemic coagulation, activation of platelets, and impaired fibrinolysis [13, 14]. F2, F5, F7, and integrin beta 3 all are crucial proteins responsible for the maintenance of hemostasis [14, 27].

Here, we found that the G/A genotype of the rs1143634 polymorphism within the *IL1B* gene, the G/T genotype of the rs3212227 polymorphism within the *IL12B* gene, the A/G genotype of the rs1130864 polymorphism within the *CRP* gene, the A/G genotype of the rs13290979 polymorphism within the *NOTCH1* gene, and the G allele of the rs1801197 polymorphism within the *CALCR* gene were associated with a decreased risk of IE whereas the T/T genotype of the rs1205 polymorphism within the *CRP* gene was associated with a higher risk of IE. Recent studies by Weinstock et al. [37] and Giannitsioti et al. [38] revealed that the SNPs within the *IL1B*, *IL6*, and *TNF* genes can be associated with IE; however, we did not confirm these findings with regard to *IL6* and *TNF*. Small sample sizes, sample differences (e.g., age, gender, ethnicity, and clinical features), and geographical variations in the microbial causes of IE [39, 40] may be responsible for these discrepancies. Since all the SNPs in our study were in Hardy-Weinberg equilibrium, genotyping errors were unlikely to affect the results.

The G/A genotype of the rs1143634 polymorphism within the *IL1B* gene and the G/T genotype of the rs3212227 polymorphism within the *IL12B* gene were associated with a higher plasma level of IL-1*β* and IL-12 that may provide an insight into their possible protective role. It has been reported that both cytokines are abundant in the serum of the patients with IE compared to those with other infections [34]; one can explain this as a specific immune response to the bacterial or fungal infection of the heart valves or chambers. We suggest that IL-1*β* and IL-12 can limit the infection, preventing further progression of IE. However, we did not find the associations of two SNPs within the *CRP* gene reaching statistical significance with CRP plasma level, although it is also known to be higher in patients with IE compared to other subjects [11]. Other SNPs within the *TNF-α*, *IL-6*, *IL-8*, and *IL-10* genes did not show a predictive value and did not correlate with altered levels of the corresponding cytokines.

Our study has a number of limitations. First, the sample size is relatively small due to the low incidence rate of IE; however, this is a common drawback of genetic association studies on IE. With our sample size (123 cases and 300 controls), we had at least 83% and 99.7% power to detect odds ratio (OR) = 2 and OR = 3, respectively, with 5% alpha risk. Unfortunately, it was still impossible to analyze the genetic associations with the features or severity of IE. Second, technical difficulties made it unable to collect information on the potential confounders, that is, alcohol consumption, smoking status, and so forth. Third, we could not properly assess the microbiological profile due to extensive antibiotic therapy of IE at the district hospitals prior to the admission to our clinic. Finally, this study included only those patients requiring surgical treatment, as other patients with IE are not admitted to our clinic due to peculiarities of Russian healthcare.

## 5. Conclusions

Inherited variation within the cytokine, acute phase response, and calcium metabolism genes can be linked to IE, providing additional insight into its pathogenesis. In particular, heterozygous genotypes of the rs1143634 and rs3212227 polymorphisms are associated with both decreased risk of IE and higher level of IL-1*β* and IL-12, respectively, suggesting their possible importance. Further studies are needed to confirm our findings and for the further understanding of the genetic susceptibility to IE.

## Supplementary Material

Supplementary Figure 1. Measurement of plasma tumor necrosis factor-α and interleukin-6 in patients with infective endocarditis at the hospital admission and 7 days postoperation. Two-tailed Student's t-test with the further Tukey's post hoc test to adjust for multiple comparisons, each dot is a measure from one patient, n.s. is for not significant. Supplementary Figure 2. Measurement of plasma interleukin-8 and interleukin-10 in patients with infective endocarditis at the hospital admission and 7 days postoperation. Two-tailed Student's t-test with the further Tukey's post hoc test to adjust for multiple comparisons, each dot is a measure from one patient, n.s. is for not significant.

## Figures and Tables

**Table 1:** Characteristics of the study population.

	Controls	Cases	Total	*p* value
Number of subjects	300 (70.92%)	123 (29.07%)	423 (100.00%)	
Median age with 95% CI	55.00 (53.00–56.00)	50.00 (48.00–53.00)	53.00 (52.00–55.00)	0.12
Interquartile range	44–62	37–59	42–61	
Males	190 (63.00%)	77 (63.00%)	267 (63.12%)	0.99
Females	110 (37.00%)	46 (37.00%)	156 (36.88%)	

CI: confidence interval.

**Table 2:** Characteristics of the patients with infective endocarditis.

*Basic characteristics*		
*Type of infective endocarditis*	*Location*	*Valve involved*
Native (93/123, 75.61%)	Left-sided (116/123, 94.31%)	Aortic (45/123, 36.58%)
		Mitral (62/123, 50.41%)
		Aortic and mitral (9/123, 7.31%)
Prosthetic (30/123, 24.39%)	Right-sided (6/123, 4.88%)	Tricuspid (6/123, 4.88%)
Device (0/123, 0.0%)	Double-sided (1/123, 0.81%)	Mitral and tricuspid (1/123, 0.81%)
*Echocardiography characteristics (mean ± standard deviation)*		
Left atrial diameter, cm	4.78 ± 0.90	
Left ventricular end-diastolic diameter, cm	6.01 ± 1.17	
Left ventricular end-systolic diameter, cm	3.92 ± 0.76	
Left ventricular end-diastolic volume, cm^3^	207.80 ± 93.98	
Left ventricular end-systolic volume, cm^3^	72.13 ± 36.05	
Interventricular septal thickness, cm	1.06 ± 0.18	
Left ventricular posterior wall thickness, cm	1.07 ± 0.19	
Left ventricular ejection fraction, %	64.00 ± 7.64	
Right atrial diameter, cm	5.20 ± 1.19	
Right ventricular diameter, cm	2.51 ± 1.08	
*Hospital complications*		
Hydrothorax	32/123 (26.02%)	
Pneumonia	21/123 (17.07%)	
Multiple organ dysfunction syndrome	17/123 (13.82%)	
Arrhythmia	33/123 (26.83%)	
Heart block	7/123 (5.69%)	
Heart failure	46/123 (37.40%)	
Myocardial infarction	4/123 (3.25%)	
Stroke	2/123 (1.63%)	
*Hospital case fatality rate*		
Death	1/123 (0.81%)	

**Table 3:** Features of the gene polymorphisms used in the study.

Single nucleotide polymorphism	Nucleotide substitution	Chromosomal position	Amino acid substitution	Forward 5′-3′ and reverse 3′-5′ polymerase chain reaction primers
*IL1B* gene				
rs1143634	G > A	113590390	Phe105Phe	F: cataagcctcgttatcccatgtgtc R: aagaagataggttctgaaatgtgga
*IL6* gene				
rs1554606	T > G	22768707	Intronic	F: ttagttcatcctgggaaaggtactc R: cagggccttttccctctctggctgc
rs1800796	G > C	22766246	5′-upstream	F: atggccaggcagttctacaacagcc R: ctcacagggagagccagaacacaga
rs2069827	G > T	22765456	5′-upstream	F: gcccaacagaggtcactgttttatc R: atcttgaagagatctcttcttagca
*IL6R* gene				
rs2228145	A > T/C	154426970	Asp358Val/Ala	F: aattttttttttaacctagtgcaag R: ttcttcttcagtaccactgcccaca
rs2229238	T > C	154437896	3′-UTR	F: ccagcagcctggaccctgtggatga R: aaaacacaaacgggctcagcaaaag
*IL8* gene				
rs2227306	C > T	74607055	Intronic	F: aactctaactctttatataggaagt R: gttcaatgttgtcagttatgactgt
*IL10* gene				
rs1800871	A > G	206946634	5′-upstream	F: agtgagcaaactgaggcacagagat R: ttacatcacctgtacaagggtacac
rs1800872	T > G	206946407	5′-upstream	F: ttttactttccagagactggcttcctacag R: acaggcggggtcacaggatgtgttccaggc
rs1800896	T > C	206946897	5′-upstream	F: tcctcttacctatccctacttcccc R: tcccaaagaagccttagtagtgttg
*IL12B* gene				
rs3212227	T > G	158742950	3′-UTR	F: attgtttcaatgagcatttagcatc R: aactatacaaatacagcaaagatat
*IL12RB* gene				
rs375947	A > G	18180451	Met365Thr	F: aggctgccattcaatgcaatacgtc R: tgctctgagcccgggctggccaata
*TNF* gene				
rs361525	G > A	31543101	5′-upstream	F: ggcccagaagacccccctcggaatc R: gagcagggaggatggggagtgtgag
rs1800629	G > A	31543031	5′-upstream	F: gaggcaataggttttgaggggcatg R: ggacggggttcagcctccagggtcc
*CRP* gene				
rs3093077	A > C	159679636	Not announced	F: ggaatccaggcaagtacgacaaccc R: tctgagactagtgggcagttgtcct
rs1130864	G > A	159683091	3′-UTR	F: cctcaaattctgattcttttggacc R: tttcccagcatagttaacgagctcc
rs1205	C > T	159682233	3′-UTR	F: acttccagtttggcttctgtcctca R: agtctctctccatgtggcaaacaag
*APOB* gene				
rs1042031	C > T	21225753	Glu4181Lys	F: caatcagatgcttgactttcatatggaatt R: ttgagtaactcgtaccaagccatcaaacac
rs6725189	G > T	21219001	Not announced	F: ttcccagcctcagctcaacagagctatggg R: cagcagtcggccctctctattgttctttcc
*APOE* gene				
rs7412	C > T	45412079	Arg176Cys	F: ctcctccgcgatgccgatgacctgcagaag R: gcctggcagtgtaccaggccggggcccgcg
rs429358	T > C	45411941	Cys130Arg	F: gcccggctgggcgcggacatggaggacgtg R: gcggccgcctggtgcagtaccgcggcgagg
*LIPC* gene				
rs1800588	C > T	58723675	5′-upstream	F: tctttgcttcttcgtcagctccttttgaca R: gggggtgaagggttttctgcaccacacttt
*LPA* gene				
rs10455872	A > G	161010118	Intronic	F: tcagacaccttgttctcagaaccca R: tgtgtttatacaggttagaggagaa
*NOTCH1* gene				
rs13290979	A > G	139425634	Intronic	F: ccagcccagcagtgaagaaactgagcccac R: accctcctggcctgacctacactcgggctt
*VDR* gene				
rs2228570	A > G	48272895	Met1Thr/Lys/Arg	F: ggcagggaagtgctggccgccattgcctcc R: tccctgtaagaacagcaagcaggccacggt
*CASR* gene				
rs1042636	A > G	122003769	Arg990Gly	F: gatgagcctcagaagaacgccatggcccac R: ggaattctacgcaccagaactccctggagg
*OPG* gene				
rs3134069	A > C	119964988	5′-upstream	F: ggagcttcctacgcgctgaacttctggagt R: gcctcctcgaggtctttccactagcctcaa
rs2073618	G > C	119964052	Asn3Lys	F: gggacttaccacgagcgcgcagcacagcaa R: ttgttcattgtggtccccggaaacctcagg
rs3102735	T > C	119965070	5′-upstream	F: ctttgctctagggttcgctgtctcccccat R: aattccctggtctagaagttagacttgatg
*CALCR* gene				
rs1801197	A > G	93055753	Leu481Pro	F: tcgccttggttgttggctggttcattcctc R: gctcctgatggcagatgtaaattgggatgt
*F2* gene				
rs1799963	G > A	46761055	3′-UTR	F: gttcccaataaaagtgactctcagc R: agcctcaatgctcccagtgctattc
*F5* gene				
rs6025	T > C	169519049	Gln534Arg	F: ttacttcaaggacaaaatacctgtattcct R: gcctgtccagggatctgctcttacagatta
rs6027	T > C	169483561	Asp2222Gly	F: gggtttttgaatgttcaattctagtaaata R: cacagccaaagagttccaggcgaagtgcaa
*F7* gene				
rs6046	G > A	113773159	Arg412Gln/Pro/Leu	F: acagtggaggcccacatgccacccactacc R: gggcacgtggtacctgacgggcatcgtcag
*ITGB3* gene				
rs5918	T > C	45360730	Leu59Pro	F: tttgggctcctgacttacaggccctgcctc R: gggctcacctcgctgtgacctgaaggagaa

IL: interleukin; TNF: tumor necrosis factor; CRP: C-reactive protein; APO: apolipoprotein; LIPC: hepatic lipase; LPA: lipoprotein (a); VDR: vitamin D receptor; CASR: calcium-sensing receptor; OPG: osteoprotegerin; CALCR: calcitonin receptor; ITGB: integrin beta.

**Table 4:** Association of the polymorphisms within the cytokine immunity genes, acute phase response genes, hemostasis genes, genes of lipid metabolism, and genes of calcium metabolism with infective endocarditis.

Model	Genotype	Without infective endocarditis	With infective endocarditis	OR (95% CI)	*p* value	AIC	HWE
*IL1B* rs1143634							
Codominant	G/G	154 (51.3%)	82 (67.8%)	1.00	0.0029	472.5	0.89
	G/A	123 (41%)	28 (23.1%)	0.43 (0.26–0.72)			
	A/A	23 (7.7%)	11 (9.1%)	0.97 (0.43–2.18)			
Dominant	G/G	154 (51.3%)	82 (67.8%)	1.00	0.0036	473.8	
	G/A-A/A	146 (48.7%)	39 (32.2%)	0.51 (0.32–0.81)			
Recessive	G/G-G/A	277 (92.3%)	110 (90.9%)	1.00	0.52	481.8	
	A/A	23 (7.7%)	11 (9.1%)	1.30 (0.59–2.88)			
Overdominant	G/G-A/A	177 (59%)	93 (76.9%)	1.00	0.0016	470.6	
	G/A	123 (41%)	28 (23.1%)	0.43 (0.26–0.71)			
Log-additive	—	—	—	0.70 (0.48–1.00)	0.046	478.2	
*IL6* rs1554606							
Codominant	G/G	92 (30.7%)	31 (25.4%)	1.00	0.31	483	0.99
	G/T	149 (49.7%)	62 (50.8%)	1.38 (0.82–2.33)			
	T/T	59 (19.7%)	29 (23.8%)	1.57 (0.84–2.95)			
Dominant	G/G	92 (30.7%)	31 (25.4%)	1.00	0.15	481.2	
	G/T–T/T	208 (69.3%)	91 (74.6%)	1.44 (0.87–2.36)			
Recessive	G/G-G/T	241 (80.3%)	93 (76.2%)	1.00	0.36	482.5	
	T/T	59 (19.7%)	29 (23.8%)	1.28 (0.76–2.17)			
Overdominant	G/G-T/T	151 (50.3%)	60 (49.2%)	1.00	0.58	483	
	G/T	149 (49.7%)	62 (50.8%)	1.13 (0.73–1.76)			
Log-additive	—	—	—	1.26 (0.92–1.72)	0.14	481.2	
*IL6* rs1800796							
Codominant	G/G	260 (86.7%)	104 (85.2%)	1.00	0.98	485.3	0.64
	C/G	38 (12.7%)	17 (13.9%)	1.07 (0.56–2.02)			
	C/C	2 (0.7%)	1 (0.8%)	1.17 (0.08–17.14)			
Dominant	G/G	260 (86.7%)	104 (85.2%)	1.00	0.83	483.3	
	C/G-C/C	40 (13.3%)	18 (14.8%)	1.07 (0.57–2.00)			
Recessive	G/G-C/G	298 (99.3%)	121 (99.2%)	1.00	0.91	483.3	
	C/C	2 (0.7%)	1 (0.8%)	1.16 (0.08–16.99)			
Overdominant	G/G-C/C	262 (87.3%)	105 (86.1%)	1.00	0.85	483.3	
	C/G	38 (12.7%)	17 (13.9%)	1.06 (0.56–2.02)			
Log-additive	—	—	—	1.07 (0.60–1.92)	0.82	483.3	
*IL6* rs2069827							
Codominant	G/G	245 (81.7%)	99 (81.2%)	1.00	0.82	484.9	0.51
	G/T	51 (17%)	22 (18%)	1.20 (0.68–2.14)			
	T/T	4 (1.3%)	1 (0.8%)	0.93 (0.10–8.66)			
Dominant	G/G	245 (81.7%)	99 (81.2%)	1.00	0.55	483	
	G/T–T/T	55 (18.3%)	23 (18.9%)	1.19 (0.68–2.08)			
Recessive	G/G-G/T	296 (98.7%)	121 (99.2%)	1.00	0.93	483.3	
	T/T	4 (1.3%)	1 (0.8%)	0.90 (0.10–8.33)			
Overdominant	G/G-T/T	249 (83%)	100 (82%)	1.00	0.53	482.9	
	G/T	51 (17%)	22 (18%)	1.20 (0.68–2.14)			
Log-additive	—	—	—	1.15 (0.69–1.92)	0.6	483.1	
*IL6R* rs2228145							
Codominant	A/A	144 (48%)	62 (50.8%)	1.00	0.46	483.8	0.89
	C/A	127 (42.3%)	45 (36.9%)	0.84 (0.52–1.34)			
	C/C	29 (9.7%)	15 (12.3%)	1.32 (0.64–2.73)			
Dominant	A/A	144 (48%)	62 (50.8%)	1.00	0.71	483.2	
	C/A-C/C	156 (52%)	60 (49.2%)	0.92 (0.59–1.43)			
Recessive	A/A-C/A	271 (90.3%)	107 (87.7%)	1.00	0.32	482.3	
	C/C	29 (9.7%)	15 (12.3%)	1.43 (0.72–2.87)			
Overdominant	A/A-C/C	173 (57.7%)	77 (63.1%)	1.00	0.32	482.3	
	C/A	127 (42.3%)	45 (36.9%)	0.79 (0.51–1.25)			
Log-additive	—	—	—	1.03 (0.74–1.43)	0.85	483.3	
*IL6R* rs2229238							
Codominant	C/C	158 (52.7%)	62 (50.8%)	1.00	0.48	483.8	0.88
	C/T	121 (40.3%)	47 (38.5%)	1.00 (0.63–1.60)			
	T/T	21 (7%)	13 (10.7%)	1.63 (0.73–3.61)			
Dominant	C/C	158 (52.7%)	62 (50.8%)	1.00	0.69	483.2	
	C/T–T/T	142 (47.3%)	60 (49.2%)	1.09 (0.71–1.70)			
Recessive	C/C-C/T	279 (93%)	109 (89.3%)	1.00	0.22	481.8	
	T/T	21 (7%)	13 (10.7%)	1.63 (0.75–3.51)			
Overdominant	C/C-T/T	179 (59.7%)	75 (61.5%)	1.00	0.78	483.3	
	C/T	121 (40.3%)	47 (38.5%)	0.94 (0.60–1.47)			
Log-additive	—	—	—	1.16 (0.82–1.63)	0.4	482.6	
*IL8* rs2227306							
Codominant	C/C	100 (33.3%)	49 (40.2%)	1.00	0.42	483.6	0.99
	C/T	147 (49%)	59 (48.4%)	0.83 (0.52–1.33)			
	T/T	53 (17.7%)	14 (11.5%)	0.63 (0.31–1.28)			
Dominant	C/C	100 (33.3%)	49 (40.2%)	1.00	0.29	482.2	
	C/T–T/T	200 (66.7%)	73 (59.8%)	0.78 (0.50–1.23)			
Recessive	C/C-C/T	247 (82.3%)	108 (88.5%)	1.00	0.28	482.2	
	T/T	53 (17.7%)	14 (11.5%)	0.70 (0.37–1.35)			
Overdominant	C/C-T/T	153 (51%)	63 (51.6%)	1.00	0.79	483.3	
	C/T	147 (49%)	59 (48.4%)	0.94 (0.61–1.46)			
Log-additive	—	—	—	0.80 (0.58–1.12)	0.19	481.6	
*IL10* rs1800871							
Codominant	G/G	183 (61%)	69 (56.6%)	1.00	0.88	485.1	0.61
	A/G	105 (35%)	47 (38.5%)	1.12 (0.71–1.78)			
	A/A	12 (4%)	6 (4.9%)	0.98 (0.34–2.81)			
Dominant	G/G	183 (61%)	69 (56.6%)	1.00	0.66	483.1	
	A/G-A/A	117 (39%)	53 (43.4%)	1.11 (0.71–1.73)			
Recessive	G/G-A/G	288 (96%)	116 (95.1%)	1.00	0.9	483.3	
	A/A	12 (4%)	6 (4.9%)	0.94 (0.33–2.64)			
Overdominant	G/G-A/A	195 (65%)	75 (61.5%)	1.00	0.61	483.1	
	A/G	105 (35%)	47 (38.5%)	1.13 (0.71–1.77)			
Log-additive	—	—	—	1.06 (0.73–1.55)	0.74	483.2	
*IL10* rs1800872							
Codominant	G/G	185 (62.1%)	67 (55.4%)	1.00	0.55	481.3	0.86
	T/G	101 (33.9%)	49 (40.5%)	1.27 (0.80–2.01)			
	T/T	12 (4%)	5 (4.1%)	0.86 (0.28–2.61)			
Dominant	G/G	185 (62.1%)	67 (55.4%)	1.00	0.39	479.8	
	T/G-T/T	113 (37.9%)	54 (44.6%)	1.22 (0.78–1.90)			
Recessive	G/G-T/G	286 (96%)	116 (95.9%)	1.00	0.65	480.3	
	T/T	12 (4%)	5 (4.1%)	0.78 (0.26–2.34)			
Overdominant	G/G-T/T	197 (66.1%)	72 (59.5%)	1.00	0.29	479.4	
	T/G	101 (33.9%)	49 (40.5%)	1.28 (0.81–2.01)			
Log-additive	—	—	—	1.11 (0.76–1.63)	0.57	480.2	
*IL10* rs1800896							
Codominant	T/T	88 (29.3%)	34 (28.1%)	1.00	0.82	481.7	0.35
	T/C	157 (52.3%)	66 (54.5%)	1.17 (0.70–1.95)			
	C/C	55 (18.3%)	21 (17.4%)	1.17 (0.59–2.29)			
Dominant	T/T	88 (29.3%)	34 (28.1%)	1.00	0.53	479.7	
	T/C-C/C	212 (70.7%)	87 (71.9%)	1.17 (0.71–1.91)			
Recessive	T/T–T/C	245 (81.7%)	100 (82.6%)	1.00	0.87	480	
	C/C	55 (18.3%)	21 (17.4%)	1.05 (0.59–1.88)			
Overdominant	T/T-C/C	143 (47.7%)	55 (45.5%)	1.00	0.66	479.9	
	T/C	157 (52.3%)	66 (54.5%)	1.10 (0.71–1.72)			
Log-additive	—	—	—	1.09 (0.78–1.52)	0.61	479.8	
*IL12B* rs3212227							
Codominant	T/T	191 (63.7%)	86 (70.5%)	1.00	0.076	480.2	0.86
	G/T	96 (32%)	29 (23.8%)	0.57 (0.34–0.96)			
	G/G	13 (4.3%)	7 (5.7%)	1.20 (0.45–3.24)			
Dominant	T/T	191 (63.7%)	86 (70.5%)	1.00	0.067	480	
	G/T-G/G	109 (36.3%)	36 (29.5%)	0.64 (0.40–1.04)			
Recessive	T/T-G/T	287 (95.7%)	115 (94.3%)	1.00	0.5	482.9	
	G/G	13 (4.3%)	7 (5.7%)	1.41 (0.53–3.75)			
Overdominant	T/T-G/G	204 (68%)	93 (76.2%)	1.00	0.025	478.3	
	G/T	96 (32%)	29 (23.8%)	0.57 (0.34–0.94)			
Log-additive	—	—	—	0.78 (0.53–1.16)	0.21	481.8	
*IL12RB* rs375947							
Codominant	A/A	137 (45.7%)	53 (43.4%)	1.00	0.58	484.3	0.60
	A/G	135 (45%)	55 (45.1%)	1.18 (0.74–1.89)			
	G/G	28 (9.3%)	14 (11.5%)	1.44 (0.68–3.03)			
Dominant	A/A	137 (45.7%)	53 (43.4%)	1.00	0.37	482.5	
	A/G-G/G	163 (54.3%)	69 (56.6%)	1.23 (0.79–1.91)			
Recessive	A/A-A/G	272 (90.7%)	108 (88.5%)	1.00	0.45	482.7	
	G/G	28 (9.3%)	14 (11.5%)	1.32 (0.65–2.68)			
Overdominant	A/A-G/G	165 (55%)	67 (54.9%)	1.00	0.66	483.1	
	A/G	135 (45%)	55 (45.1%)	1.10 (0.71–1.72)			
Log-additive	—	—	—	1.19 (0.85–1.67)	0.3	482.3	
*TNF* rs361525							
Codominant	G/G	274 (91.3%)	112 (91.8%)	1.00	0.81	484.9	0.46
	A/G	25 (8.3%)	10 (8.2%)	0.90 (0.40–1.99)			
	A/A	1 (0.3%)	0 (0%)	0.00 (0.00–NA)			
Dominant	G/G	274 (91.3%)	112 (91.8%)	1.00	0.74	483.2	
	A/G-A/A	26 (8.7%)	10 (8.2%)	0.88 (0.39–1.94)			
Recessive	G/G-A/G	299 (99.7%)	122 (100%)	1.00	0.55	483	
	A/A	1 (0.3%)	0 (0%)	0.00 (0.00–NA)			
Overdominant	G/G-A/A	275 (91.7%)	112 (91.8%)	1.00	0.79	483.3	
	A/G	25 (8.3%)	10 (8.2%)	0.90 (0.40–2.00)			
Log-additive	—	—	—	0.86 (0.39–1.87)	0.7	483.2	
*TNF* rs1800629							
Codominant	G/G	229 (76.3%)	97 (79.5%)	1.00	0.66	484.5	0.99
	A/G	67 (22.3%)	24 (19.7%)	0.89 (0.51–1.54)			
	A/A	4 (1.3%)	1 (0.8%)	0.41 (0.04–3.93)			
Dominant	G/G	229 (76.3%)	97 (79.5%)	1.00	0.56	483	
	A/G-A/A	71 (23.7%)	25 (20.5%)	0.85 (0.50–1.46)			
Recessive	G/G-A/G	296 (98.7%)	121 (99.2%)	1.00	0.42	482.7	
	A/A	4 (1.3%)	1 (0.8%)	0.42 (0.04–4.02)			
Overdominant	G/G-A/A	233 (77.7%)	98 (80.3%)	1.00	0.71	483.2	
	A/G	67 (22.3%)	24 (19.7%)	0.90 (0.52–1.55)			
Log-additive	—	—	—	0.83 (0.51–1.37)	0.47	482.8	
*CRP* rs3093077							
Codominant	C/C	262 (87.3%)	112 (92.6%)	1.00	0.28	481.5	0.99
	A/C	37 (12.3%)	8 (6.6%)	0.55 (0.24–1.23)			
	A/A	1 (0.3%)	1 (0.8%)	1.99 (0.12–34.12)			
Dominant	C/C	262 (87.3%)	112 (92.6%)	1.00	0.17	480.2	
	A/C-A/A	38 (12.7%)	9 (7.4%)	0.59 (0.27–1.29)			
Recessive	C/C-A/C	299 (99.7%)	120 (99.2%)	1.00	0.61	481.8	
	A/A	1 (0.3%)	1 (0.8%)	2.10 (0.12–36.04)			
Overdominant	C/C-A/A	263 (87.7%)	113 (93.4%)	1.00	0.13	479.7	
	A/C	37 (12.3%)	8 (6.6%)	0.55 (0.24–1.23)			
Log-additive	—	—	—	0.67 (0.33–1.36)	0.25	480.7	
*CRP* rs1130864							
Codominant	G/G	142 (47.3%)	68 (55.7%)	1.00	0.018	477.9	0.41
	A/G	134 (44.7%)	38 (31.1%)	0.58 (0.36–0.93)			
	A/A	24 (8%)	16 (13.1%)	1.45 (0.70–3.00)			
Dominant	G/G	142 (47.3%)	68 (55.7%)	1.00	0.12	481.4	
	A/G-A/A	158 (52.7%)	54 (44.3%)	0.71 (0.45–1.09)			
Recessive	G/G-A/G	276 (92%)	106 (86.9%)	1.00	0.094	481.1	
	A/A	24 (8%)	16 (13.1%)	1.83 (0.91–3.69)			
Overdominant	G/G-A/A	166 (55.3%)	84 (68.8%)	1.00	0.0083	476.9	
	A/G	134 (44.7%)	38 (31.1%)	0.54 (0.34–0.86)			
Log-additive	—	—	—	0.93 (0.67–1.30)	0.68	483.7	
*CRP* rs1205							
Codominant	C/C	112 (37.3%)	38 (31.9%)	1.00	0.018	468.4	0.11
	C/T	154 (51.3%)	56 (47.1%)	1.06 (0.64–1.74)			
	T/T	34 (11.3%)	25 (21%)	2.50 (1.28–4.90)			
Dominant	C/C	112 (37.3%)	38 (31.9%)	1.00	0.28	473.3	
	C/T–T/T	188 (62.7%)	81 (68.1%)	1.29 (0.81–2.07)			
Recessive	C/C-C/T	266 (88.7%)	94 (79%)	1.00	0.0047	466.5	
	T/T	34 (11.3%)	25 (21%)	2.42 (1.32–4.43)			
Overdominant	C/C-T/T	146 (48.7%)	63 (52.9%)	1.00	0.34	473.6	
	C/T	154 (51.3%)	56 (47.1%)	0.80 (0.52–1.25)			
Log-additive	—	—	—	1.47 (1.05–2.05)	0.023	469.3	
*APOB* rs1042031							
Codominant	C/C	210 (70.5%)	83 (69.8%)	1.00	0.86	477.7	0.40
	C/T	78 (26.2%)	33 (27.7%)	1.00 (0.61–1.65)			
	T/T	10 (3.4%)	3 (2.5%)	0.69 (0.18–2.71)			
Dominant	C/C	210 (70.5%)	83 (69.8%)	1.00	0.88	476	
	C/T–T/T	88 (29.5%)	36 (30.2%)	0.96 (0.59–1.56)			
Recessive	C/C-C/T	288 (96.6%)	116 (97.5%)	1.00	0.59	475.7	
	T/T	10 (3.4%)	3 (2.5%)	0.69 (0.18–2.69)			
Overdominant	C/C-T/T	220 (73.8%)	86 (72.3%)	1.00	0.95	476	
	C/T	78 (26.2%)	33 (27.7%)	1.01 (0.62–1.67)			
Log-additive	—	—	—	0.94 (0.62–1.42)	0.76	475.9	
*APOB* rs6725189							
Codominant	G/G	196 (65.8%)	72 (59.5%)	1.00	0.53	481.2	0.46
	G/T	89 (29.9%)	44 (36.4%)	1.31 (0.82–2.10)			
	T/T	13 (4.4%)	5 (4.1%)	1.01 (0.33–3.04)			
Dominant	G/G	196 (65.8%)	72 (59.5%)	1.00	0.3	479.4	
	G/T–T/T	102 (34.2%)	49 (40.5%)	1.27 (0.81–2.00)			
Recessive	G/G-G/T	285 (95.6%)	116 (95.9%)	1.00	0.87	480.5	
	T/T	13 (4.4%)	5 (4.1%)	0.92 (0.31–2.73)			
Overdominant	G/G-T/T	209 (70.1%)	77 (63.6%)	1.00	0.26	479.2	
	G/T	89 (29.9%)	44 (36.4%)	1.31 (0.82–2.08)			
Log-additive	—	—	—	1.17 (0.80–1.71)	0.42	479.9	
*APOE* rs7412							
Codominant	C/C	251 (83.7%)	104 (86%)	1.00	0.63	483.2	0.71
	C/T	48 (16%)	16 (13.2%)	0.79 (0.42–1.49)			
	T/T	1 (0.3%)	1 (0.8%)	2.37 (0.13–43.65)			
Dominant	C/C	251 (83.7%)	104 (86%)	1.00	0.53	481.7	
	C/T–T/T	49 (16.3%)	17 (14.1%)	0.82 (0.44–1.53)			
Recessive	C/C-C/T	299 (99.7%)	120 (99.2%)	1.00	0.55	481.7	
	T/T	1 (0.3%)	1 (0.8%)	2.45 (0.13–45.09)			
Overdominant	C/C-T/T	252 (84%)	105 (86.8%)	1.00	0.44	481.5	
	C/T	48 (16%)	16 (13.2%)	0.78 (0.42–1.48)			
Log-additive	—	—	—	0.86 (0.48–1.56)	0.63	481.8	
*APOE* rs429358							
Codominant	T/T	239 (79.7%)	96 (78.7%)	1.00	0.71	485.2	0.99
	C/T	58 (19.3%)	24 (19.7%)	1.22 (0.70–2.14)			
	C/C	3 (1%)	2 (1.6%)	1.58 (0.24–10.40)			
Dominant	T/T	239 (79.7%)	96 (78.7%)	1.00	0.44	483.3	
	C/T-C/C	61 (20.3%)	26 (21.3%)	1.24 (0.72–2.14)			
Recessive	T/T-C/T	297 (99%)	120 (98.4%)	1.00	0.67	483.7	
	C/C	3 (1%)	2 (1.6%)	1.52 (0.23–9.97)			
Overdominant	T/T-C/C	242 (80.7%)	98 (80.3%)	1.00	0.5	483.4	
	C/T	58 (19.3%)	24 (19.7%)	1.21 (0.69–2.12)			
Log-additive	—	—	—	1.23 (0.75–2.01)	0.41	483.2	
*LIPC* rs1800588							
Codominant	C/C	173 (57.7%)	77 (63.6%)	1.00	0.2	480.8	0.52
	C/T	113 (37.7%)	41 (33.9%)	0.69 (0.43–1.11)			
	T/T	14 (4.7%)	3 (2.5%)	0.48 (0.13–1.81)			
Dominant	C/C	173 (57.7%)	77 (63.6%)	1.00	0.084	479.1	
	C/T–T/T	127 (42.3%)	44 (36.4%)	0.67 (0.43–1.06)			
Recessive	C/C-C/T	286 (95.3%)	118 (97.5%)	1.00	0.35	481.2	
	T/T	14 (4.7%)	3 (2.5%)	0.55 (0.15–2.03)			
Overdominant	C/C-T/T	187 (62.3%)	80 (66.1%)	1.00	0.16	480.1	
	C/T	113 (37.7%)	41 (33.9%)	0.72 (0.45–1.15)			
Log-additive	—	—	—	0.69 (0.46–1.04)	0.071	478.8	
*LPA* rs10455872							
—	A/A	275 (92%)	108 (91.5%)	1.00	0.86	475.8	0.99
	A/G	24 (8%)	10 (8.5%)	1.08 (0.48–2.39)			
*NOTCH1* rs13290979							
Codominant	A/A	98 (32.8%)	53 (43.8%)	1.00	0.024	476.2	0.48
	A/G	152 (50.8%)	44 (36.4%)	0.53 (0.33–0.87)			
	G/G	49 (16.4%)	24 (19.8%)	0.98 (0.53–1.82)			
Dominant	A/A	98 (32.8%)	53 (43.8%)	1.00	0.05	477.9	
	A/G-G/G	201 (67.2%)	68 (56.2%)	0.64 (0.41–1.00)			
Recessive	A/A-A/G	250 (83.6%)	97 (80.2%)	1.00	0.28	480.6	
	G/G	49 (16.4%)	24 (19.8%)	1.37 (0.78–2.41)			
Overdominant	A/A-G/G	147 (49.2%)	77 (63.6%)	1.00	0.0062	474.2	
	A/G	152 (50.8%)	44 (36.4%)	0.54 (0.34–0.84)			
Log-additive	—	—	—	0.88 (0.65–1.21)	0.44	481.1	
*VDR* rs2228570							
Codominant	G/G	94 (31.5%)	36 (29.8%)	1.00	0.19	479.1	0.81
	A/G	145 (48.7%)	51 (42.1%)	0.93 (0.55–1.57)			
	A/A	59 (19.8%)	34 (28.1%)	1.54 (0.85–2.80)			
Dominant	G/G	94 (31.5%)	36 (29.8%)	1.00	0.67	480.3	
	A/G-A/A	204 (68.5%)	85 (70.2%)	1.11 (0.69–1.79)			
Recessive	G/G-A/G	239 (80.2%)	87 (71.9%)	1.00	0.07	477.2	
	A/A	59 (19.8%)	34 (28.1%)	1.61 (0.97–2.68)			
Overdominant	G/G-A/A	153 (51.3%)	70 (57.9%)	1.00	0.25	479.2	
	A/G	145 (48.7%)	51 (42.1%)	0.77 (0.50–1.20)			
Log-additive	—	—	—	1.23 (0.91–1.66)	0.19	478.8	
*CASR* rs1042636							
Codominant	A/A	244 (81.6%)	103 (85.1%)	1.00	0.2	480.5	0.75
	A/G	52 (17.4%)	18 (14.9%)	0.71 (0.39–1.30)			
	G/G	3 (1%)	0 (0%)	0.00 (0.00–NA)			
Dominant	A/A	244 (81.6%)	103 (85.1%)	1.00	0.19	480	
	A/G-G/G	55 (18.4%)	18 (14.9%)	0.67 (0.37–1.23)			
Recessive	A/A-A/G	296 (99%)	121 (100%)	1.00	0.16	479.8	
	G/G	3 (1%)	0 (0%)	0.00 (0.00–NA)			
Overdominant	A/A-G/G	247 (82.6%)	103 (85.1%)	1.00	0.28	480.5	
	A/G	52 (17.4%)	18 (14.9%)	0.72 (0.39–1.32)			
Log-additive	—	—	—	0.65 (0.37–1.17)	0.14	479.6	
*OPG* rs3134069							
Codominant	A/A	248 (83.2%)	110 (90.9%)	1.00	0.19	479.2	0.71
	A/C	49 (16.4%)	10 (8.3%)	0.53 (0.25–1.11)			
	C/C	1 (0.3%)	1 (0.8%)	1.92 (0.09–41.36)			
Dominant	A/A	248 (83.2%)	110 (90.9%)	1.00	0.1	477.9	
	A/C-C/C	50 (16.8%)	11 (9.1%)	0.56 (0.28–1.15)			
Recessive	A/A-A/C	297 (99.7%)	120 (99.2%)	1.00	0.65	480.3	
	C/C	1 (0.3%)	1 (0.8%)	2.06 (0.09–44.96)			
Overdominant	A/A-C/C	249 (83.6%)	111 (91.7%)	1.00	0.078	477.4	
	A/C	49 (16.4%)	10 (8.3%)	0.53 (0.25–1.11)			
Log-additive	—	—	—	0.62 (0.32–1.21)	0.15	478.4	
*OPG* rs2073618							
Codominant	C/C	76 (25.4%)	32 (26.7%)	1.00	0.17	478.3	0.56
	C/G	155 (51.8%)	51 (42.5%)	0.79 (0.46–1.36)			
	G/G	68 (22.7%)	37 (30.8%)	1.32 (0.73–2.41)			
Dominant	C/C	76 (25.4%)	32 (26.7%)	1.00	0.86	479.9	
	C/G-G/G	223 (74.6%)	88 (73.3%)	0.96 (0.58–1.58)			
Recessive	C/C-C/G	231 (77.3%)	83 (69.2%)	1.00	0.089	477	
	G/G	68 (22.7%)	37 (30.8%)	1.54 (0.94–2.52)			
Overdominant	C/C-G/G	144 (48.2%)	69 (57.5%)	1.00	0.098	477.2	
	C/G	155 (51.8%)	51 (42.5%)	0.69 (0.44–1.07)			
Log-additive	—	—	—	1.16 (0.85–1.58)	0.35	479	
*OPG* rs3102735							
Codominant	T/T	218 (73.2%)	95 (78.5%)	1.00	0.36	480.4	0.82
	C/T	73 (24.5%)	25 (20.7%)	0.89 (0.52–1.52)			
	C/C	7 (2.4%)	1 (0.8%)	0.25 (0.03–2.27)			
Dominant	T/T	218 (73.2%)	95 (78.5%)	1.00	0.46	480	
	C/T-C/C	80 (26.9%)	26 (21.5%)	0.82 (0.49–1.39)			
Recessive	T/T-C/T	291 (97.7%)	120 (99.2%)	1.00	0.17	478.6	
	C/C	7 (2.4%)	1 (0.8%)	0.26 (0.03–2.32)			
Overdominant	T/T-C/C	225 (75.5%)	96 (79.3%)	1.00	0.74	480.4	
	C/T	73 (24.5%)	25 (20.7%)	0.91 (0.53–1.56)			
Log-additive	—	—	—	0.78 (0.49–1.26)	0.3	479.4	
*CALCR* rs1801197							
Codominant	A/A	140 (46.8%)	76 (62.8%)	1.00	0.0077	474	0.22
	A/G	136 (45.5%)	40 (33.1%)	0.52 (0.33–0.84)			
	G/G	23 (7.7%)	5 (4.1%)	0.36 (0.13–1.03)			
Dominant	A/A	140 (46.8%)	76 (62.8%)	1.00	0.0024	472.5	
	A/G-G/G	159 (53.2%)	45 (37.2%)	0.50 (0.32–0.79)			
Recessive	A/A-A/G	276 (92.3%)	116 (95.9%)	1.00	0.13	479.5	
	G/G	23 (7.7%)	5 (4.1%)	0.48 (0.17–1.33)			
Overdominant	A/A-G/G	163 (54.5%)	81 (66.9%)	1.00	0.018	476.2	
	A/G	136 (45.5%)	40 (33.1%)	0.58 (0.36–0.92)			
Log-additive	—	—	—	0.56 (0.38–0.82)	0.002	472.2	
*F2* rs1799963							
—	G/G	286 (95.7%)	118 (98.3%)	1.00	0.10	477	0.99
	A/G	13 (4.3%)	2 (1.7%)	0.32 (0.07–1.49)			
*F5* rs6025							
Codominant	C/C	286 (95.7%)	117 (97.5%)	1.00	0.32	479.4	0.14
	C/T	12 (4%)	3 (2.5%)	0.56 (0.15–2.11)			
	T/T	1 (0.3%)	0 (0%)	0.00 (0.00–NA)			
Dominant	C/C	286 (95.7%)	117 (97.5%)	1.00	0.24	478.3	
	C/T–T/T	13 (4.3%)	3 (2.5%)	0.48 (0.13–1.79)			
Recessive	C/C-C/T	298 (99.7%)	120 (100%)	1.00	0.23	478.2	
	T/T	1 (0.3%)	0 (0%)	0.00 (0.00–NA)			
Overdominant	C/C-T/T	287 (96%)	117 (97.5%)	1.00	0.38	478.9	
	C/T	12 (4%)	3 (2.5%)	0.57 (0.15–2.13)			
Log-additive	—	—	—	0.47 (0.14–1.58)	0.19	477.9	
*F5* rs6027							
Codominant	T/T	254 (85%)	104 (86.7%)	1.00	0.35	479.5	0.12
	C/T	41 (13.7%)	16 (13.3%)	1.00 (0.52–1.91)			
	C/C	4 (1.3%)	0 (0%)	0.00 (0.00–NA)			
Dominant	T/T	254 (85%)	104 (86.7%)	1.00	0.79	479.6	
	C/T-C/C	45 (15.1%)	16 (13.3%)	0.92 (0.48–1.74)			
Recessive	T/T-C/T	295 (98.7%)	120 (100%)	1.00	0.15	477.5	
	C/C	4 (1.3%)	0 (0%)	0.00 (0.00–NA)			
Overdominant	T/T-C/C	258 (86.3%)	104 (86.7%)	1.00	0.98	479.6	
	C/T	41 (13.7%)	16 (13.3%)	1.01 (0.53–1.93)			
Log-additive	—	—	—	0.86 (0.47–1.56)	0.61	479.4	
*F7* rs6046							
Codominant	G/G	236 (78.9%)	88 (72.7%)	1.00	0.06	477.8	0.77
	A/G	59 (19.7%)	33 (27.3%)	1.58 (0.94–2.64)			
	A/A	4 (1.3%)	0 (0%)	0.00 (0.00–NA)			
Dominant	G/G	236 (78.9%)	88 (72.7%)	1.00	0.14	479.2	
	A/G-A/A	63 (21.1%)	33 (27.3%)	1.48 (0.89–2.46)			
Recessive	G/G-A/G	295 (98.7%)	121 (100%)	1.00	0.1	478.8	
	A/A	4 (1.3%)	0 (0%)	0.00 (0.00–NA)			
Overdominant	G/G-A/A	240 (80.3%)	88 (72.7%)	1.00	0.074	478.2	
	A/G	59 (19.7%)	33 (27.3%)	1.60 (0.96–2.68)			
Log-additive	—	—	—	1.32 (0.82–2.14)	0.26	480.1	
*ITGB3* rs5918							
Codominant	T/T	214 (71.6%)	88 (73.3%)	1.00	0.5	480.2	0.66
	C/T	77 (25.8%)	26 (21.7%)	0.84 (0.49–1.43)			
	C/C	8 (2.7%)	6 (5%)	1.69 (0.55–5.19)			
Dominant	T/T	214 (71.6%)	88 (73.3%)	1.00	0.76	479.5	
	C/T-C/C	85 (28.4%)	32 (26.7%)	0.93 (0.56–1.52)			
Recessive	T/T-C/T	291 (97.3%)	114 (95%)	1.00	0.32	478.6	
	C/C	8 (2.7%)	6 (5%)	1.77 (0.58–5.39)			
Overdominant	T/T-C/C	222 (74.2%)	94 (78.3%)	1.00	0.45	479	
	C/T	77 (25.8%)	26 (21.7%)	0.82 (0.48–1.38)			
Log-additive	—	—	—	1.02 (0.68–1.54)	0.92	479.6	

All the ORs and 95% CIs are adjusted for age and gender. IL: interleukin; TNF: tumor necrosis factor; CRP: C-reactive protein; APO: apolipoprotein; LIPC: hepatic lipase; LPA: lipoprotein (a); VDR: vitamin D receptor; CASR: calcium-sensing receptor; OPG: osteoprotegerin; CALCR: calcitonin receptor; ITGB: integrin beta; OR: odds ratio; CI: confidence interval; AIC: Akaike information criterion; HWE: Hardy-Weinberg equilibrium.
