# Supplementary material for: Inherited Variation in Cytokine, Acute Phase Response, and Calcium Metabolism Genes Affects Susceptibility to Infective Endocarditis
Source: Mediators Inflamm. 2017 Jun 4;2017:7962546. doi: 10.1155/2017/7962546 (PMC5474236; doi:10.1155/2017/7962546)

**Supplementary Figure 1. Measurement of plasma tumor necrosis factor-α and interleukin-6 in patients with infective endocarditis at the hospital admission and 7 days postoperation. Two-tailed Student’s t-test with the further Tukey’s post hoc test to adjust for multiple comparisons, each dot is a measure from one patient, n.s. is for not significant**


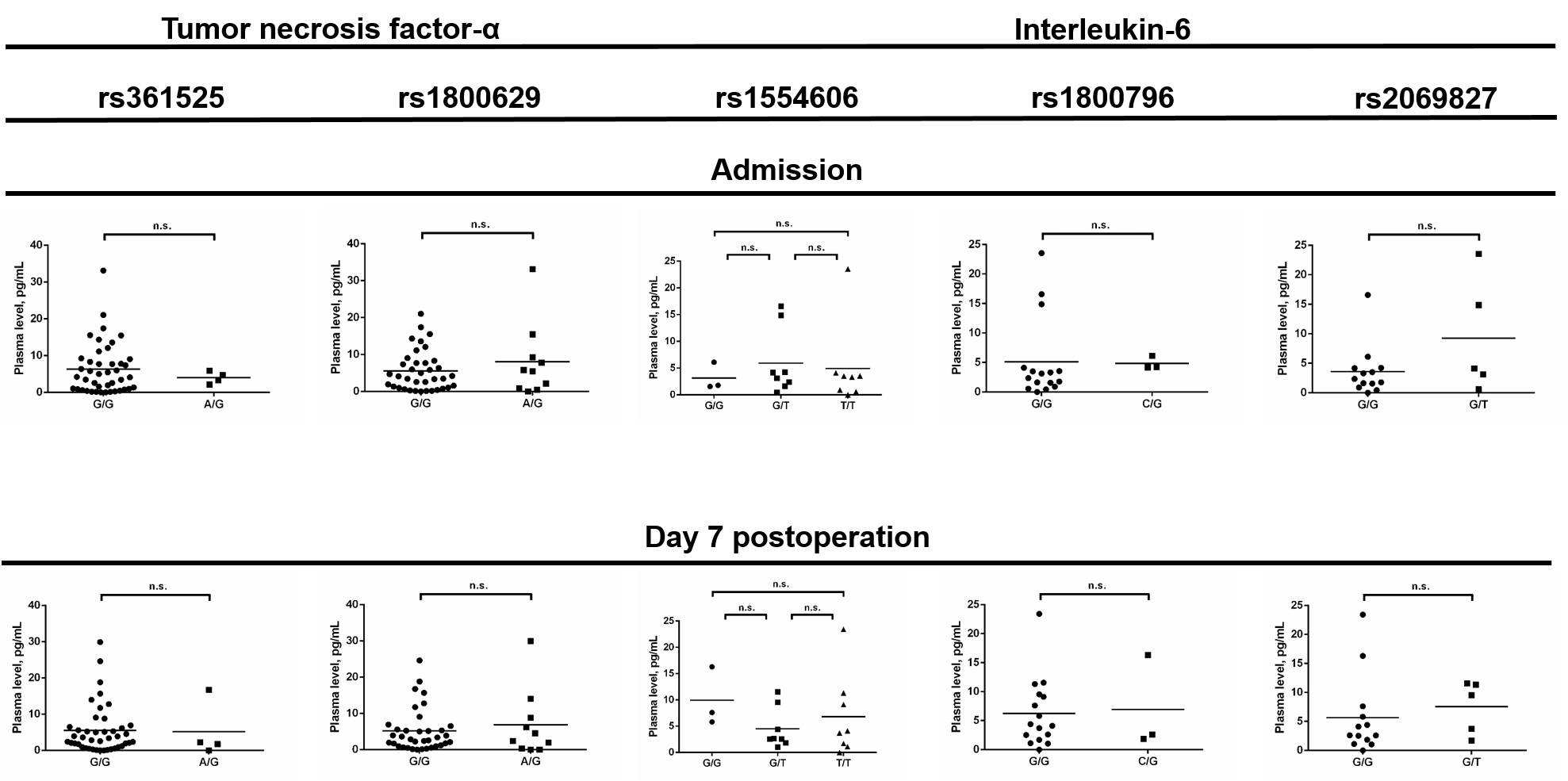

Supplement: Supplementary file 1 — Supplementary Figure 1. Measurement of plasma tumor necrosis factor-α and interleukin-6 in patients with infective endocarditis at the hospital admission and 7 days postoperation. Two-tailed Student's t-test with the further Tukey's post hoc test to adjust for multiple comparisons, each dot is a measure from one patient, n.s. is for not significant. Supplementary Figure 2. Measurement of plasma interleukin-8 and interleukin-10 in patients with infective endocarditis at the hospital admission and 7 days postoperation. Two-tailed Student's t-test with the further Tukey's post hoc test to adjust for multiple comparisons, each dot is a measure from one patient, n.s. is for not significant. [file 7962546.f1.docx]
